# Supplementary material for: Significant Microbial Changes Are Evident in the Reproductive Tract of Pregnant Rhesus Monkeys at Mid-Gestation but Their Gut Microbiome Does Not Shift until Late Gestation
Source: Microorganisms. 2023 Jun 1;11(6):1481. doi: 10.3390/microorganisms11061481 (PMC10304935; doi:10.3390/microorganisms11061481)
Supplement: Supplementary file 1 [file microorganisms-11-01481-s001.zip › microorg-2366291-supplementary.pdf]

Supplementary Materials: Methods

Subjects

Vaginal and fecal specimens were collected from female rhesus monkeys (*Macaca mulatta).* They were born and raised in a large breeding colony that had been established over 50 years ago [31]. The 72 females were healthy, multiparous adults in the breeding program. Because the photoperiod was controlled at 16 hours light/16 hours dark, with lights on at 0600, and other husbandry practices were standardized, the inherent tendency of this species to be a seasonal breeder was not expressed [32]. Ambient room temperature was also regulated and maintained at an average 21 ^{o}C. Mating and conceptions could be scheduled year-round. Thus, it was possible to obtain specimens contemporaneously from nonpregnant females with regular menstrual cycles as well as gravid and nursing females in each study. The monkeys were all fed the same diet manufactured specifically for primates (Lab Diet 5LFD, St Louis, MO), approximately 250 g of biscuits daily, and supplemented with fruit and vegetables (150 g) 3–4 times per week. Their stainless-steel cages were cleaned daily and completely sanitized with disinfectant every 2 weeks.

Breeding Strategy

A time-mating protocol was used to generate pregnant females [31]. Menstrual bleeding, which occurred monthly, was monitored. When monkeys were optimally fertile at mid-cycle, a single female was introduced to the cage of an unrelated adult male for one week. Thus, it was possible to delineate the date of conception to this one-week period and to schedule sample collection for the mid- and late-gestation conditions (i.e., at 3–4 months gestational age for Study 1a and 1b, and 3 weeks before the end of the 170-day pregnancy for Study 2, respectively). Similarly, fecal specimen collection from the cycling females in Study 2 was scheduled either 2 weeks after the last menses (i.e., follicular phase) or >2 weeks post-menses for the luteal phase. Samples from lactating females in Studies 1a and 1b were collected 2 months postpartum, which corresponds to the peak of nursing. All specimens were obtained in the morning while monkeys were momentarily immobilized in a cage with a movable side panel designed for humane handling. All samples from each study were analyzed as a single batch.

DNA Isolation and Sequencing for Study 1

Vaginal and rectal swab DNA were isolated using the PowerSoil DNA isolation kit (MoBio, Carlsbad, CA) in Study 1a. DNA samples were purified and quantified with an Invitrogen Qubit 2/4 Fluorometer (Life Technologies, Carlsbad, CA) and dsDNA High Sensitivity Assay Kit (ThermoFisher Scientific, Waltham, MA) and stored at -20 °C in 10mM Tris buffer until sequenced. DNA samples were sent to Biosciences Division Environmental Sample Preparation and Sequencing Facility (ESPSF) at the Argonne National Laboratory (Argonne, IL) for sequencing. PCR primers 515f/806r were used to amplify the variable region 4 of the 16S rRNA gene. 16S rRNA libraries were created

using Illumina-tag PCR reactions with the DNA extracts generated by the Earth Microbiome Project's protocol. Sequencing was performed using the Illumina MiSeq platform with library preparation according to the Earth Microbiome Project's protocol. DNA isolation, library preparation, and 16S rRNA gene amplicon sequencing for Study 1b were carried out with identical methods. Aliquots of the same bacterial DNA from the fecal specimens were independently analyzed by CosmosID (Rockville, MD) using metagenomic HiSeq 2x 100 bp sequencing to confirm the lack of significant changes in the gut microbiota. The purified DNA extracts were sequenced using an Illumina HiSeq platform with a paired-end 100 bp single-read configuration.

16S rRNA Gene Amplicon Data Processing and Analyses for Study 1

All 16S rRNA data processing and analyses were carried out in-house using identical data pipelines in QIIME2-2022.2. Sequences were filtered by truncating forward and reverse reads to exclude poor quality sequence reads. QIIME2's VSEARCH was used to dereplicate and identify sequences by closed-reference clustering and MAFFT-FastTree alignment and phylogenetic tree construction. The Greengenes (v.13_8) 97%-identity datasets were used as a reference and unmatched sequences were discarded. The feature classifier classify-consensus-vsearch function in QIIME2 was used for taxonomic identification, with reference to the Greengenes database. Taxa with low abundance (less than 10 reads across all samples) and low prevalence (present in less than 2 samples) were filtered out to reduce noise in the dataset. Linear discriminant analysis Effect Size (LEfSe) was used for the multi-level comparison of taxa across reproductive phases. Alpha and beta diversity analyses were conducted in QIIME2, and diversity plots were generated using the ggplot2 package in R. Four alpha diversity indices (Pielou's index, Faith's Phylogenetic Diversity, Observed Species, and Shannon index) were generated, significances of difference were ascertained with Kruskal–Wallis tests, and beta diversity was analyzed using both weighted and unweighted UniFrac distance matrices. Comparison tests included PERMANOVA (if PERMDISP $p \geq 0.05$), ANOSIM (if PERMDISP $p \leq 0.05$), and post hoc pairwise comparisons using ANOSIM with 999 permutations. The Kyoto Encyclopedia of Genes and Genomes (KEGG) functional pathways were inferred for Study 1 data using the Phylogenetic Investigation of Communities by Reconstruction of Unobserved States (PICRUSt) tool from the bioBakery suite. Statistical comparisons of KEGG functional pathways were performed using false discovery rate (FDR)-adjusted analysis of variance (ANOVA) in R.

Metagenomic Data Processing and Analyses for Study 1b

In addition to the 16S rRNA gene amplicon sequencing in Studies 1a and 1b, metagenomic data were generated using the GENIUS software package (CosmosID, Inc., Rockville, MD), as described previously for both human and nonhuman primate

samples [55-58]. Briefly, the CosmosID-HUB Microbiome uses raw, unassembled reads as input and matches the sequences against a curated reference database derived from private and public databases of assembled genomes. GenBook™ is comprised of millions of marker sequences representing both coding and non-coding sequences shared or uniquely identified across taxonomic or phylogenetic levels. The results are presented to the researcher in tabular format for further analysis, including taxonomic names, OTU IDs, frequency, and relative abundance. To determine if there were significant shifts in the most abundant phyla and genera, taxa were ranked and relative abundances in the hindgut at mid-gestation were compared to cycling and nursing females by *a priori* t tests. Illustrations of relative abundances at the phylogenetic levels of phylum and genus are provided in Supplementary Materials—Results (Supplementary Figure S1 and Supplementary Figure S2)

DNA Extraction and Library Preparation for Study 2

Fecal specimens collected for Study 2 were sent to a different commercial vendor for analysis (Wright Labs, Huntingdon, PA). DNA was extracted using a DNeasy Powersoil Kit (Qiagen, Germantown, MD), following the manufacturer's protocol, and DNA was eluted using 50 uL of DNase/RNase-free water. Purified DNA was quantified with an Invitrogen Qubit 4 Fluorometer and 1x dsDNA High Sensitivity Assay Kit (ThermoFisher Scientific, Waltham, MA). Briefly, 16S rRNA libraries were created using Illumina-tag PCR reactions with the DNA extracts generated by the Earth Microbiome Project's protocol. PCR products were pooled and gel-purified on 2% agarose gel using the Qiagen Gel Extraction Kit (Qiagen, Germantown, MD). Before sequencing, the purified pool was quality-checked using an Agilent 2100 BioAnalyzer and Agilent DNA High Sensitivity DNA kit (Agilent Technologies, Santa Clara, CA). The purified pool was stored at -20 °C and then sequenced using Illumina MiSeq v2 chemistry with paired-end 250-base-pair reads. Metagenomic libraries were prepared using DNA extracts and the Nextera XT DNA Library Preparation kit (Illumina, San Diego, CA). Libraries were quality-checked using an Agilent 2100 Bioanalyzer and DNA High Sensitivity kit and then pooled in an equimolar ratio. The pool was gel-purified using a 2% agarose gel and the Qiagen QIAquick gel extraction kit (Qiagen, Germantown, MD). Following purification, the pool was sequenced using an Illumina NextSeq to yield 2x150 bp reads.

16S rRNA Gene Amplicon Data Processing and Analysis for Study 2

16S rRNA gene amplicon data processing was carried out in-house using the same pipelines as employed in Study 1, generating bacterial abundances, indices of alpha diversity, and beta diversity metrics, as described previously.

Metagenomic Analysis for Study 2

Read quality was checked in the raw data with fastqc. Based on initial quality, fastp was used to filter the data with a sliding window of 4, and an average Phred q score of

28 was used. Sequences shorter than 90 bp were discarded. The remaining sequences were then annotated using Kraken2 with a version of its standard database that included fungi in addition to the standard libraries. A table of species-level annotations was created for use with downstream analyses, with *Homo sapiens* being excluded to ensure that human contamination did not impact the results. HUMAnN2 was run on the sequences identified by Kraken2 using the "--bypass-prescreen" option so that the entire database was used to annotate sequences. UniRef90 gene annotations were regrouped to KEGG Orthologs (KO), and the resulting table was used for the downstream functional analysis. LEfSe was employed to identify taxonomic features with relative abundances that differed during pregnancy. Only taxa identified as having a differential abundance with a log LDA score of more than 2.0 were considered significantly enriched (Kruskal–Wallis, significance set at $p \leq 0.05$). Sparse Partial Least-Squares Discriminant Analysis (sPLS-DA) was performed using the mixOmics package through R (R Core Team 2018) to determine if coherent clusters of bacterial genes were evident for each reproductive phase from the normalized tables.

Progesterone Assay

To consider the possible influence of a key pregnancy hormone that has been implicated as a mediator of microbiome change [30], fecal and blood levels of progesterone were determined by mass spectrometry in Study 2. Specimens from cycling and pregnant females underwent extraction and analysis on a QTRAP 5500 quadruple linear ion trap mass spectrometer (AB Sciex, Framingham, MA), equipped with an atmospheric pressure chemical ionization source. The system included two LC-20ADXR pumps and an SIL20-ACXR autosampler (Shimadzu, Kyoto, Japan). Additional details about these LC-MS/MS assay methods have already been published [62, 63].

Supplementary Materials: Results

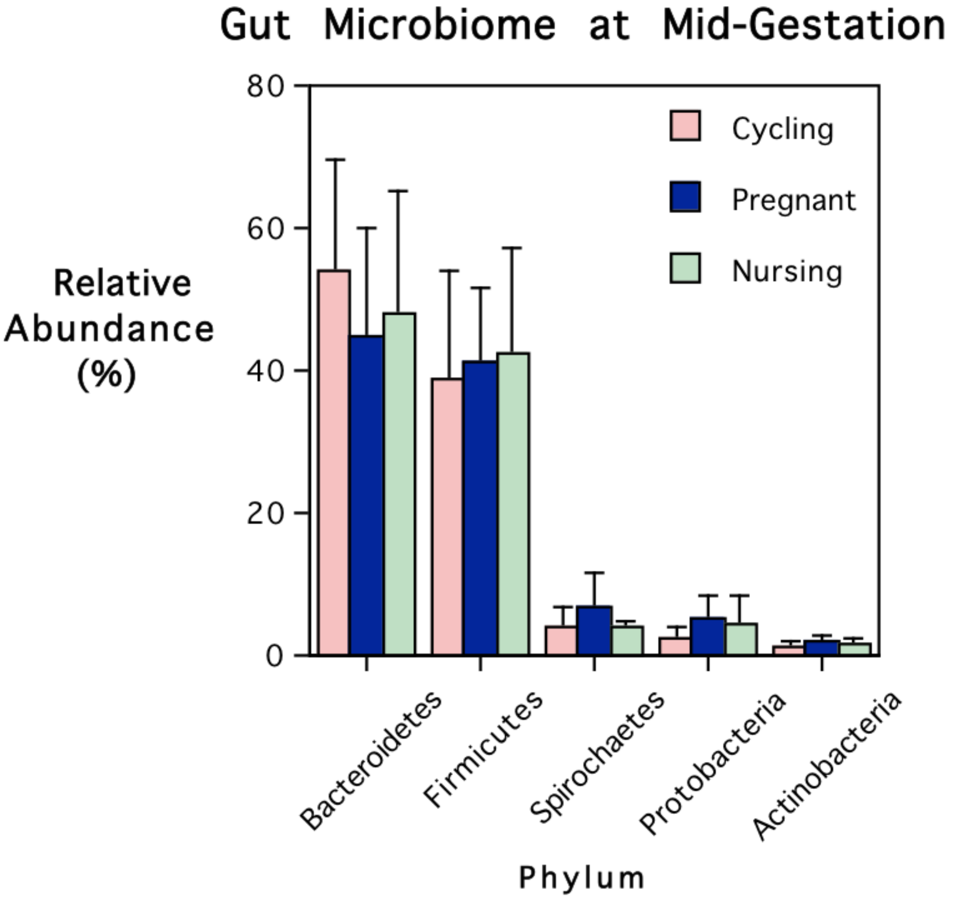

Supplementary Figure S1. The relative abundances of 5 major phyla in fecal specimens from rhesus monkeys at mid-gestation were not significantly different when compared to cycling and nursing females in Study 1b. *Bacteroidetes* and *Firmicutes* were the predominant phyla in all monkeys (48.8 % and 40.7%, respectively), and did not evince a significant shift at mid-gestation. *Spirochetes*, *Proteobacteria*, and *Actinobacteria* were present at lower levels in the hindgut of monkeys (4.8%, 3.9% and 1.4%, respectively), and the relative abundances were not significantly different at mid-gestation. Taxonomic annotation was performed with the CosmosID metagenomic platform (for more details, see Supplementary Materials: Methods). Mean (+SD) abundances are shown for the 5 phyla in each reproductive phase.

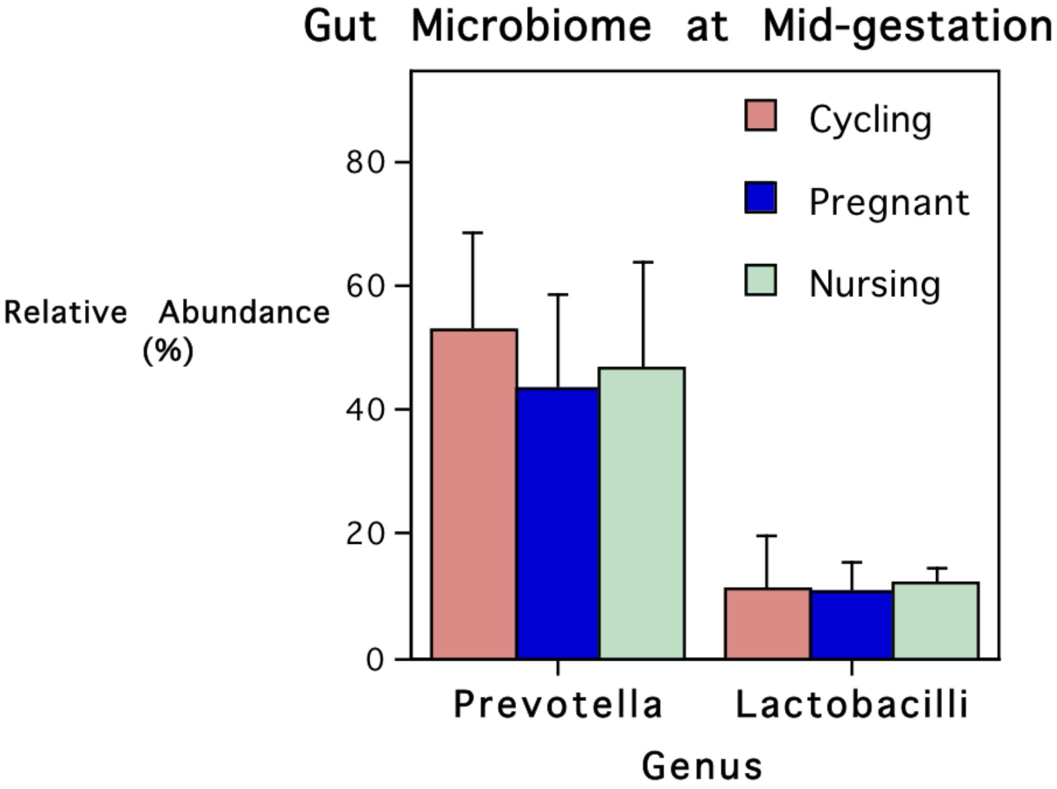

Supplementary Figure S2. The relative abundance of *Prevotella*—the most prevalent bacterial genus in the monkey hindgut—was similar at mid-gestation to the level detected in cycling and nursing females (overall mean = 47.8%). In addition, the relative abundance of lactobacilli was similar at mid-gestation to the abundance detected in cycling and nursing monkeys. Mean (+SD) levels are shown for each reproductive phase. *Bifidobacterium* were present at low levels, below 1.0%, but the abundance was more likely to be quantified above 0.05% in nursing females (8 of 11 nursing females as compared to 1 of 11 cycling and 1 of 11 pregnant female monkeys). Study 1b was conducted to replicate and extend the findings from Study 1a indicating the absence of significant change in the gut microbiome at mid-gestation. This taxonomic identification illustrated in Figure S2 was generated with the CosmosID metagenomic platform (for more details, see Supplementary Materials—Methods).

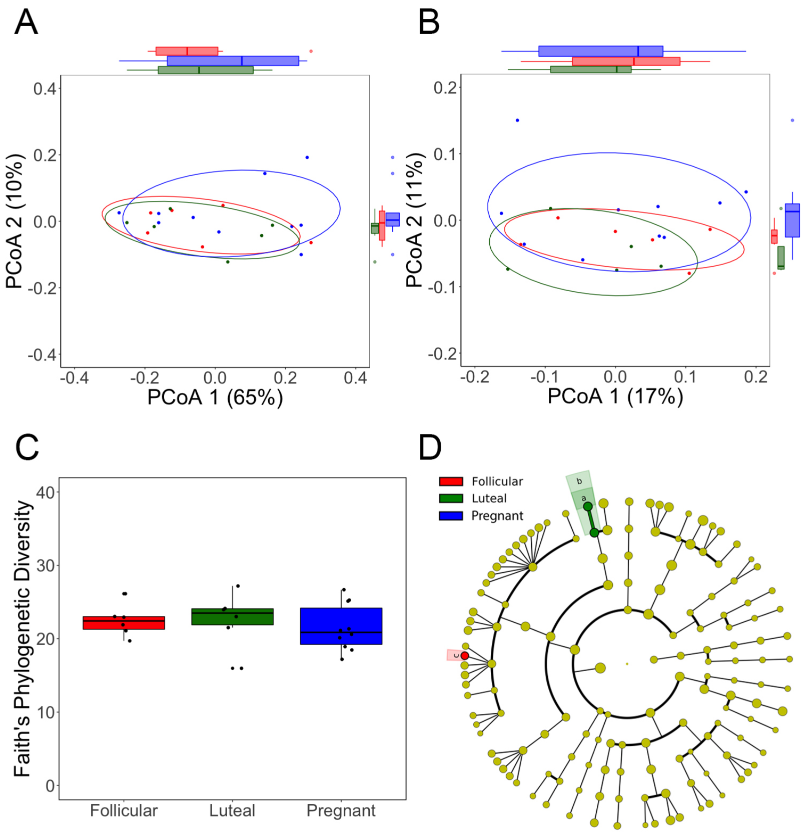

LEfSe Phylogenetic Enrichment

Follicular Taxa	LDA	Luteal Taxa	LDA	Pregnant Taxa	LDA
Anaerovibrio (g)	3.72	*Streptococcaceae (f)*	4.68	Lactobacillus reuteri (s)	4.48
		Streptococcus (g)	4.68		
		Prevotella stercorea (s)	4.62		

Top 10 bacteria sorted by LDA effect size. LDA > 2 and p ≤ 0.05 shown. Taxonomic level indicated by letters: (p) phylum, (c) class, (o) order, (f) family, (g) genus, and (s) species.

Supplementary Figure S3. The 16S rRNA gene amplicon sequencing of gut bacteria in late-gestation monkeys from Study 2 identified only one enriched taxon when compared to females in the follicular and luteal phases of the menstrual cycle. In addition, the PCoA suggested only a small and non-significant shift in beta diversity metrics but with considerable overlap in the microbial profiles (S3A, weighted; S3B, unweighted). In addition, there was no change in alpha diversity when comparing pregnant to cycling females (Panel S3C). The cladogram (S3D) illustrates the extent of the taxonomic enrichment when using 16s rRNA gene amplicon sequencing. Colored spheres on the circular lines connote taxa at different phylogenetic levels that delineated each reproductive phase (species to kingdom, innermost to outer rings, respectively).The suggestion of an increased abundance of *L. reuteri* in the hindgut of late-pregnant monkeys was also seen with metagenomic sequencing (see Suppl. Tables 5 and 6). The latter analysis enumerated a significant enrichment of *Firmicutes* at the phylum level, including several species of *Lactobacillus*. (LDA > 2 and p < 0.05; see Suppl. Figure 4 and Suppl. Table 6). The taxonomic level in the LEfSe table is indicated by small letters: phylum (p), class (c), order (o), family (f), genus (g), and species (s).

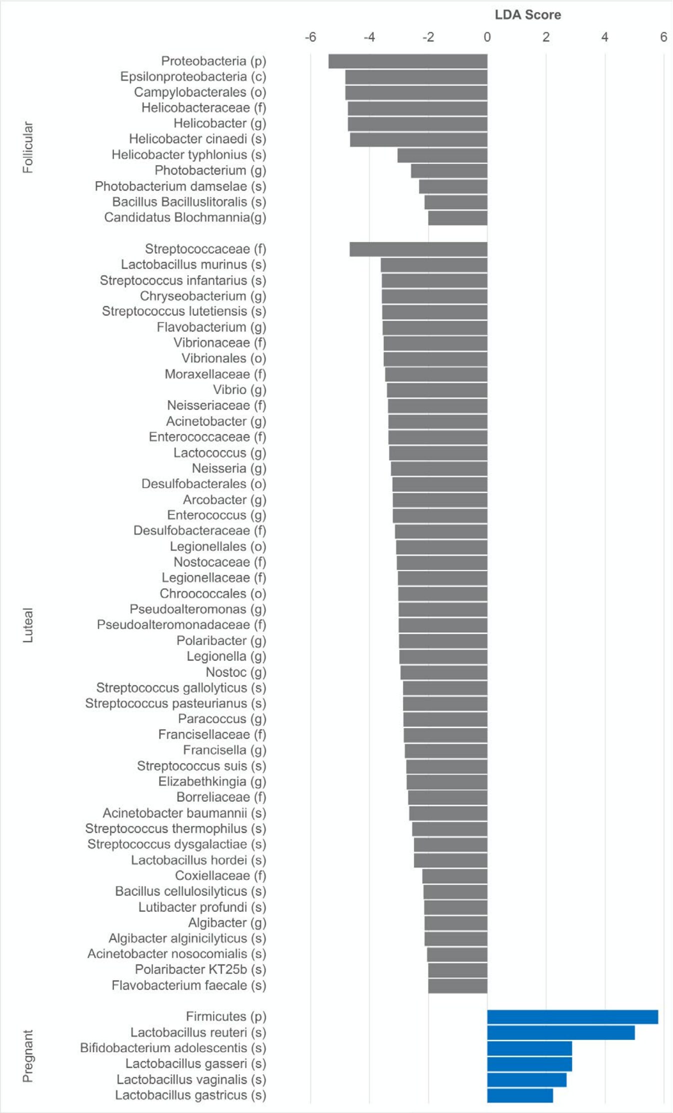

Supplementary Figure S4. Bacterial taxa with significantly different LDA scores in cycling versus late-pregnant female rhesus monkeys. This analysis was based on the metagenomic sequencing of fecal samples from 10 late-pregnant females, 3 weeks before delivery, and 12 cycling females (6 follicular and 6 luteal phase) in Study 2. Microbial differences in the hindgut of late-pregnant and cycling females are also visually illustrated as a cladogram in Figure 4A. Supplementary Figure S4 is an enlarged version of Figure 4B. Supporting values for this graphic illustration are provided in Supplementary Table S6. The genomic enrichment of microbial genes during pregnancy was examined with PCA and sPLS-DA. An analysis of clustered set of 310 genes delineating the pregnant hindgut is visually illustrated in Figure 5b. A list of specific bacterial genes that were differentially abundant in each reproductive phase is provided in Supplementary Table S7.
